# *Leptospira* Infection Interferes with the Prothrombinase Complex Assembly during Experimental Leptospirosis

**DOI:** 10.3389/fmicb.2017.00500

**Published:** 2017-03-28

**Authors:** Monica L. Vieira, Sonia A. de Andrade, Zenaide M. Morais, Silvio A. Vasconcellos, Maria Lucia Z. Dagli, Ana Lucia T. O. Nascimento

**Affiliations:** ^1^Laboratorio Especial de Desenvolvimento de Vacinas, Instituto ButantanSão Paulo, Brazil; ^2^Laboratório de Síntese Orgânica - Laboratório Especial de Toxinologia AplicadaSão Paulo, Brazil; ^3^Laboratório de Zoonoses Bacterianas, Faculdade de Medicina Veterinária e Zootecnia, Universidade de São PauloSão Paulo, Brazil; ^4^Departmento de Patologia, Faculdade de Medicina Veterinária e Zootecnia, Universidade de São PauloSão Paulo, Brazil; ^5^Programa de Pós-Graduação Interunidades em Biotecnologia, Instituto de Ciências Biomédicas, Universidade de São PauloSão Paulo, Brazil

**Keywords:** *Leptospira*, leptospirosis, coagulation, hemostasis, prothrombinase complex

## Abstract

Leptospirosis is a worldwide zoonotic and neglected infectious disease of human and veterinary concern, caused by pathogenic *Leptospira* species. Although bleeding is a common symptom of severe leptospirosis, the cause of hemorrhage is not completely understood. In severe infections, modulation of hemostasis by pathogens is an important virulence mechanism, and hemostatic impairments such as coagulation/fibrinolysis dysfunction are frequently observed. Here, we analyze the coagulation status of experimentally infected hamsters in an attempt to determine coagulation interferences and the origin of leptospirosis hemorrhagic symptomatology. Hamsters were experimentally infected with *L. interrogans*. The lungs, kidneys, and livers were collected for culture, histopathology, and coagulation assays. *L. interrogans* infection disturbs normal coagulation in the organs of animals. Our results suggest the presence of a thrombin-like factor or FX activator, which is able to activate FII in the leptospirosis organ extracts. The activity of those factors is accelerated in the prothrombinase complex. Additionally, we show for the first time that live leptospires act as a surface for the prothrombinase complex assembly. Our results contribute to the understanding of leptospirosis pathophysiological mechanisms and may open new routes for the discovery of novel treatments in the severe manifestations of the disease.

## Introduction

Leptospirosis is a neglected zoonotic bacterial disease of worldwide public health importance. It affects humans, domestic, and wildlife animals and it is caused by pathogenic bacteria of the genus *Leptospira* (Levett, 2001; Bharti et al., 2003). Human infection occurs mainly through contact with tissues or urine of wild or domestic infected animals or exposure to contaminated soil or water (Faine et al., 1999; Plank and Dean, 2000). Although underestimated, it is believed that more than 500,000 cases of symptomatic leptospirosis cases occur annually (Ko et al., 2009). Human leptospirosis incidence is low in developed countries, whereas in developing countries, the high incidence is associated to the lack of adequate sanitation as well as favorable environmental conditions to the transmission of leptospires (Haake et al., 2002; Hartskeerl et al., 2011).

Leptospires typically enter the body through the mucous membranes or through cuts on the skin, persist and multiply in the bloodstream during the leptospiremic phase of the illness, spreading into the surrounding tissue. Kidneys and liver are the preferential colonization sites. Soon after the host has developed an immunological response, bacteria are cleared from blood, characterizing the immune phase (Levett, 2001). Human infection varies from subclinical to severe manifestations, but usually presents initially as a non-specific, sudden onset illness with fever, myalgia, and headache. Signs of bleeding are also common, and occur in most patients with severe leptospirosis (Levett, 2001; Vinetz, 2001).

Hemostasis is a tightly regulated biological process that stops blood loss when a vascular injury occurs. Primary hemostasis involves the response of the vascular endothelium and platelets to blood vessel injury, resulting in the formation of a platelet plug. Secondary hemostasis reinforces the platelet plug by the deposition of insoluble fibrin, formed by the action of the thrombin generated during the proteolytic coagulation cascade. Thus, the conversion of prothrombin to thrombin is crucial for hemostasis. The specific proteolytic activation of prothrombin is catalyzed by the prothrombinase complex, which results from the reversible binding of the coagulation factor Xa (FXa) to the factor Va (FVa) in phospholipidic membranes in the presence of calcium ions. The clots formed to help to stop the bleeding are eventually dissolved through fibrinolysis. There is a delicate equilibrium between coagulation and fibrinolysis during the hemostatic process, thus any disturbance may lead to coagulopathies resulting in thrombosis or hemorrhage.

Bleeding is a common symptom of severe leptospirosis. Although the cause of leptospirosis hemorrhages is not completely understood, some studies report ongoing fibrinolysis, activation of coagulation, impaired anticoagulation, and thrombocytopenia, whereas the involvement of disseminated intravascular coagulation is controversial (Sitprija et al., 1980; Edwards et al., 1986; De Francesco Daher et al., 2002; Chierakul et al., 2008; Wagenaar et al., 2010; Vieira et al., 2016; Vieira and Nascimento, 2016). Endothelial cell infection and activation has also been implied as a mechanism in leptospirosis hemorrhages, contributing to severe disease manifestations (Martinez-Lopez et al., 2010; Goeijenbier et al., 2015). As modulation of hemostasis by pathogenic bacteria is an important virulence mechanism (Tapper and Herwald, 2000; Frick et al., 2007; Oehmcke and Herwald, 2010), we believe that coagulation/fibrinolysis dysfunction might be involved in the pathophysiological manifestation of leptospirosis. The present investigation aims to further decipher the role of coagulation and hemostatic imbalance during the disease *in vivo*, using leptospirosis hamster model.

Here, we show that *L. interrogans* infection disturbs normal coagulation in the organs of animals, observable by the prolongation of global coagulation tests. Also, our results suggest the presence of a thrombin-like factor or FXa activator able to activate FII in the leptospirosis organ extracts, which activity is accelerated in the prothrombinase complex. Furthermore, we show that live leptospires act as a surface for the prothrombinase complex assembly. Our results contribute to understanding of coagulation disorders during leptospirosis and may shed light to novel strategies to fight the disease.

## Methods

### Bacteria strains and culture conditions

*L. interrogans* serovar Kennewicki strain Pomona Fromm (LPF) were cultured at 28°C in Elinghausen-McCullough-Johnson-Harris (EMJH) medium (BD, Difco) supplemented with 10% *Leptospira* enrichment EMJH medium (BD, Difco), 0.3 g/L peptone (BD, Difco), and 0.2 g/L meat extract (Sigma-Aldrich).

### Animal experimental infection

Groups of 4–6 male Golden Syrian hamsters (8–10 weeks old) were experimentally infected intraperitoneally with the lethal dose of 10^4^ *L. interrogans* serovar Kennewicki strain Pomona Fromm (LPF)/animal in 200 μL PBS (Phosphate Buffer Saline). After 0, 3, 6, 8, 9, or 10 days post-infection (pi.), the animals were euthanized and the lungs, kidneys, and livers were aseptically collected. A control group of non-infected (ni) hamsters was maintained in the same conditions for 10 days.

### Organ-derived *Leptospira* culture

The organs were aseptically washed twice with PBS, macerated in PBS and inoculated onto semi-solid EMJH medium. The samples were maintained at 28°C and the growth of leptospires in the cultures was monitored for up to 40 days. The cultures were considered positive by visual determination and dark-field microscopy confirmation.

### Organ extracts processing

After collection, the lungs, kidneys and livers were immediately washed twice with PBS. Organ fragments were added to 10 μL/mg lysis buffer (150 mM sodium chloride, 1% Triton X-100, 0.1% sodium-dodecyl sulfate, 50 mM Tris-HCl pH 7.4). After maceration by pestle and mortar, the samples were centrifuged to debris removal. The protein contents of the extracts were determined by Bradford assay.

### Histopathological analysis

Representative tissue samples were collected from the kidneys, liver, and lungs of the animals. Tissue sections were fixed in 10% neutral formalin and routinely embedded in paraffin. Tissue sections (2–4 μm) were stained with haematoxylin-eosin (H-E) and examined by an experienced pathologist.

### Clotting assays

Prothrombin time (PT), thrombin clotting time (TCT), and activated partial thromboplastin time (aPTT) were measured by standard procedures (Knights and Ingram, 1967). The results were expressed in seconds and the maximum time of the test was 300 s. The tests were made in duplicate and the results were expressed as averages of each sample determination.

### Prothrombin F1+2 fragments assay

The F1+2 fragments contents of the organ extracts were determined by using the kit Enzygnost F1+2 (Dade Behring) according to the manufacturer instructions. Twenty microliters of the samples at 2 mg/mL were used for each replicate.

### Preparation of the phospholipidic membranes

The phosphatidylserine/phosphatidylcholine (PSPC; Sigma) membranes were prepared as described elsewhere (Barenholz et al., 1977).

### Prothrombinase complex assay

The complex was formed by mixing 8 nM FXa (Hyphen Biomed), 5 nM FVa (Sigma), 10 nM CaCl_2_, and 3 μM PCPS (75:25 mol/mol) in buffer containing 0.05 M Tris/HCl, 150 mM NaCl, pH 7.4. The mixture was incubated for 30 min at 37°C with or without the addition of the organ extracts (100 ng/μL), followed by addition of 20 nM prothrombin (FII; Hyphen Biomed) and 1 mM of the thrombin-specific chromogenic substrate S-2238 (Hyphen Biomed). The formation of thrombin was determined absorbance determination at 405 nm at different time points. In the blank reaction, FII was omitted.

Alternatively, experiments were performed without the addition of FXa or FII. We also analyzed the formation of the complex by substituting the PCPS by live leptospires (5 × 10^7^/mL).

### FXa and thrombin activity assays

Leptospires (10^8^, 5 × 10^7^, and 10^7^/mL) were lysed by gentle sonication in buffer containing 0.05 M Tris/HCl, 10 nM CaCl_2_, 150 mM NaCl, pH 7.4. After addition of 1 mM chromogenic substrate S-2238 or S-2765 (Hyphen Biomed) for thrombin and FXa activities, respectively, the extracts were incubated for 16 h at 37°C and the optical densities (ODs) were measured at 405 nm.

### Ethics statement

All animal studies were approved by the Ethical Committee for Animal Research of Instituto Butantan and of School of Veterinary Medicine and Animal Science, University of Sao Paulo, Sao Paulo, SP, Brazil under the protocols 798/11 and 3158/13, respectively. The Committees in Animal Research adopt the guidelines of the Brazilian College of Animal Experimentation.

## Results

### Hamster infection

Groups of hamsters were experimentally infected intraperitoneally with a lethal dose of *L. interrogans*. After 0, 3, 6, 8, 9, or 10 days, the animals were euthanized and the lungs, kidneys and livers were collected for culture, histopathology, and coagulation assays. Although not initially planned, the group euthanized at day 9 was included because the animals were severely sick and would not survive until the day 10. A group of non-infected hamsters was maintained throughout the experiment and exhibited no signs of disease.

In the infected hamsters, there was a rapid migration of *Leptospira* to the liver, as all animals presented positive culture as early as day 3 pi. In the kidneys and lungs, the bacterial load progressively increased, peaking on days 6–8 pi. After that, we noted a gradual clearance of the leptospires from lungs and livers, whereas there seems to be a bacterial persistence in kidneys (Table 1).

**Table 1:** **Presence of viable *Leptospira* in the organs of experimentally infected hamsters**.

**Days post-infection**	**Lungs**	**Kidneys**	**Livers**
0	0/6	0/6	0/6
3	2/6	3/6	6/6
6	5/6	5/6	5/6
8	3/6	5/6	3/6
9	3/4	3/4	3/4
10	0/5	3/5	1/5
10 (ni)	0/6	0/6	0/6

*The animals were infected with a lethal dose of L. interrogans serovar Kennewicki strain Pomona Fromm (LPF). After 0, 3, 6, 8, 9, or 10 days post-infection, the animals were euthanized and the lungs, kidneys, and livers were aseptically collected. A control group of non-infected (ni) hamsters was maintained in the same conditions for 10 days. The organs were processed and inoculated into semi-solid EMJH medium. The growth of leptospires in the cultures were monitored for up to 40 days*.

**Figure 1:**
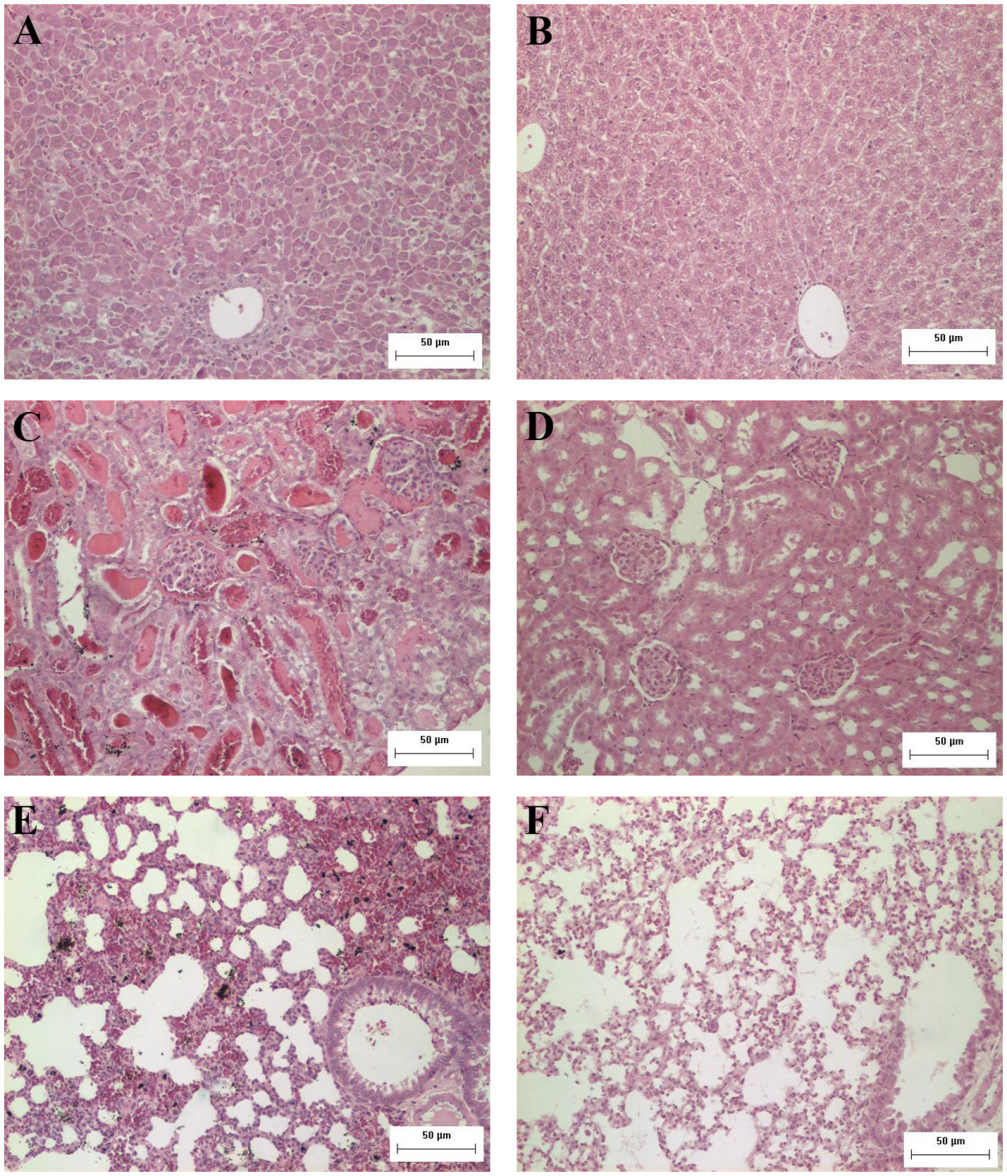
**Representative photomicrographs from histopathological findings in organs from hamsters experimentally infected with *Leptospira***. **(A)** Hamster liver 8 days after inoculation with *L. interrogans*: observe the disorganization and loss of normal trabecular architecture; **(B)** control liver; **(C)** hamster kidney 8 days after inoculation with *L. interrogans*: observe tubular nephrosis and necrosis, with the presence of hyaline casts; **(D)** control kidney; **(E)** hamster lung 8 days after inoculation with *L. interrogans*: observe the intense congestion and hemorrhage; **(F)** control lung. H&E. Bar = 50 μm.

The histopathological analysis of organs from the infected group showed significant and progressive lesions accompanying the evolution of the disease, while groups at day 0 pi. and non-infected showed only discrete congestion. The observed lesions are characteristic of leptospirosis, and are described in Table 2. Noteworthy, intense congestion and hemorrhage were observed in lungs, while nephrosis and necrosis were observed in the kidneys of the severely sick animals. The livers showed the typical loss of normal trabecular architecture pattern observed in leptospirosis (Figure 1).

**Table 2:** **Histopathological analysis of organs from hamsters experimentally infected with *Leptospira***.

**Organ**	**Days post-infection**	**Histopathological observation**
Lung	0	Discrete congestion
	3	Congestion, discrete widened alveolar septa
	6	Congestion, discrete hemorrhage, widened alveolar septa, proliferation of bronchial epithelium
	8	Congestion, multiple foci of widened alveolar septa, hemorrhage
	9	Discrete congestion, diffuse widened alveolar septa (interstitial pneumonia), proliferation of bronchial epithelium
	10	Congestion, intense hemorrhage, interstitial pneumonia, hyperplasia, hypertrophy of bronchial epithelium
	10 (ni)	Discrete congestion
Kidney	0	Discrete congestion
	3	Discrete congestion
	6	Congestion, discrete hemorrhage
	8	Congestion, hemorrhage, foci of nephrosis, and tubular necrosis
	9	Intense congestion, intense hemorrhage, nephrosis, and tubular necrosis
	10	Intense congestion, intense hemorrhage, intense tubular necrosis
	10 (ni)	Discrete congestion
Liver	0	Discrete congestion
	3	Discrete focal and periportal hepatitis
	6	Congestion, discrete hemorrhage, cytoplasmic rarefaction in hepatocytes
	8	Congestion, foci of hepatitis, vacuolar degeneration, degradation of trabecular architecture, discrete hepatocyte necrosis
	9	Congestion, hemorrhage, degradation of trabecular architecture, focal hepatitis
	10	Congestion, focal and periportal hepatitis, hepatocyte cytoplasmic rarefaction
	10 (ni)	Discrete congestion

*The animals were infected with a lethal dose of L. interrogans serovar Kennewicki strain Pomona Fromm (LPF). After 0, 3, 6, 8, 9, or 10 days post-infection, the animals were euthanized and the lungs, kidneys, and livers were removed and prepared for histopathology by H-E staining. Organs from non-infected (ni) hamsters were used as controls*.

### Coagulation times

**Figure 2:**
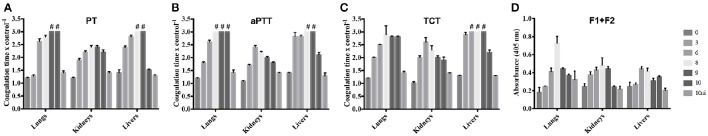
**Leptospirosis organ extracts prolong coagulation times and contain higher levels of prothrombin F1+2 fragments**. Lungs, kidneys, and livers of experimentally infected hamsters were removed after 0, 3, 6, 8, 9, or 10 days pi. Organs from a non-infected group (10 ni) were used as controls. The organs extracts were mixed with human citrated plasma and the PT **(A)**, aPTT **(B)**, and TCT **(C)** were determined in a coagulometer. Plasma alone was used as control in those experiments. **(D)** Depicts the quantification of prothrombin F1+2 fragments in the organ extracts. The bars represent the mean of the ratio samples/control plasma ± standard deviation of triplicates. ^#^Ratio sample/control plasma > 3.

In an attempt to better determine the origin of the leptospirosis hemorrhages, we prepared extracts of the lungs, kidneys, and livers of infected animals and control group. The extracts were assayed for their ability to interfere with the normal clotting of human plasma. The extracts of all organs prolonged the PT, denoting an interference with the extrinsic coagulation cascade, as well as the aPTT, interfering with the intrinsic or common pathways (Figures 2A,B). The TCT was also prolonged, showing that the final steps of coagulation were also compromised (Figure 2C). A stronger interference was observed with lung and liver extracts in all the assays when compared to extracts from kidneys. The ability to prolong the clotting times was dependent on the evolution of the disease. In some assays, the coagulation interference of samples from animals at day 10 pi. was lower than the day 9 pi. This can be explained by the fact that the animals euthanized at day 9 pi. were severely sick and would not survive until the day 10, thus the euthanasia was anticipated. To further assess the coagulation activation, we measured prothrombin F1+2 fragments within the organ samples. The F1+2 fragments contents were higher during the progression of the disease, being the peak observed at days 6 and 8 pi. (Figure 2D). The results are in line with the coagulation times experiments, corroborating the coagulation activation observation.

### Prothrombinase complex

**Figure 3:**
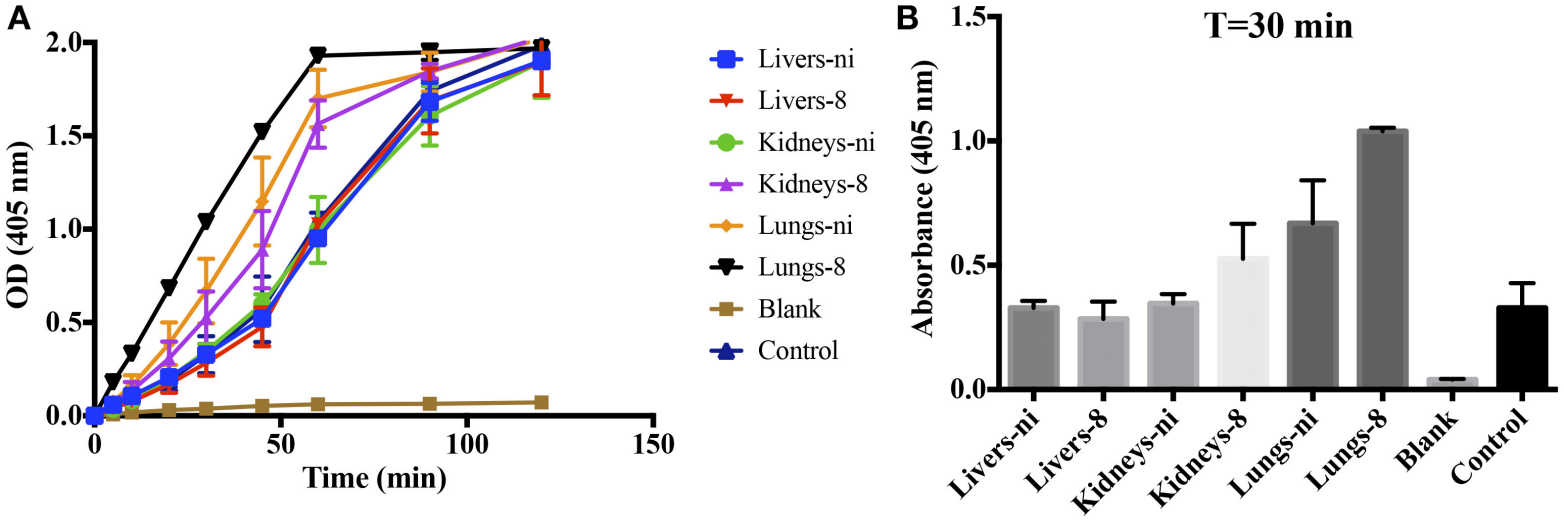
**Interference of leptospirosis organ extracts on the activity of prothrombinase complex**. Lungs, kidneys, and livers extracts of *Leptospira*-infected hamsters at day 8 or non-infected (ni.) animals were mixed with FXa, FVa, and PCPS in buffer containing calcium. After 30 min incubation at 37°C, FII was added and its conversion to thrombin was measured as cleavage of the chromogenic substrate (*OD* = 405 nm) at different time points **(A)**. In **(B)**, the bars represent the absorbance obtained 30 min after FII addition. The values represent the mean ± standard deviation of triplicates and are representative of two independent experiments.

Prothrombinase complex is comprised of FXa, FVa, and FII assembled on a phospholipid surface in the presence of Ca^2+^. Prothrombinase is the cornerstone of the coagulation cascade; the complex is positioned in such a manner that both the intrinsic and extrinsic pathways support its function and production, culminating in the production of thrombin. As our previous results showed that the organ extracts of infected hamsters prolonged both PT, aPTT, and TCT, we decided to evaluate if any interference could occur in the formation and activity of the complex. We chose to evaluate animals in day 8 pi. in comparison to non-infected ones. As shown in Figure 3, when extracts of the lungs and kidneys from animals in day 8 were added to the prothrombinase complex mixture, acceleration in the formation of the complex occurred. An effect was also observed with the lungs from ni. animals, although significantly lower than from day 8. No interference was observed when liver samples (day 8 or ni.) or kidneys from ni. animals were added to prothrombinase complex mixture when compared to the control (prothrombinase complex with no additions).

### Coagulation factors activities

**Figure 4:**
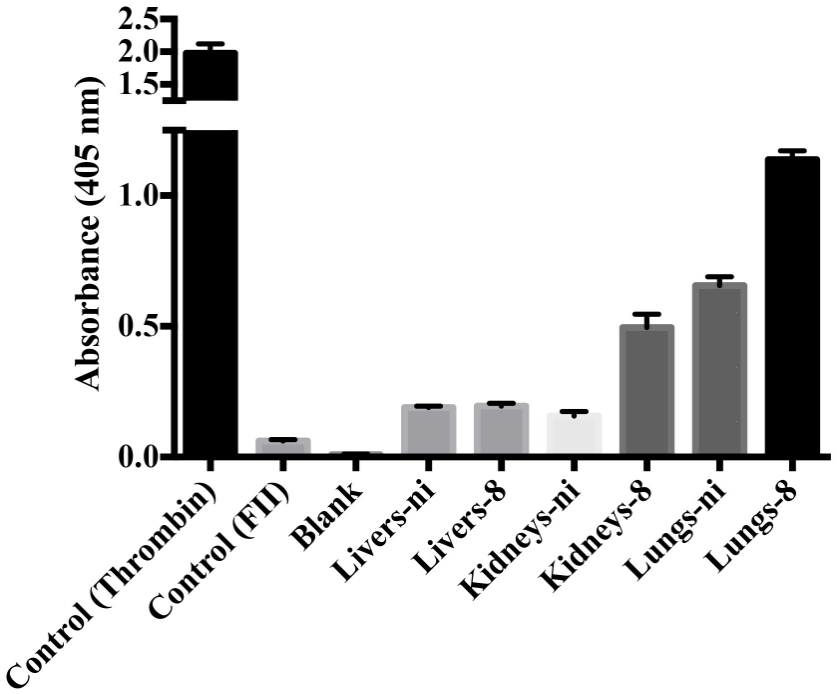
**Activity of leptospirosis organ extracts on the conversion of FII to thrombin**. Lungs, kidneys, and livers extracts of *Leptospira*-infected hamsters at day 8 or non-infected (ni.) animals were mixed with FII and incubated at 37°C for 16 h. The generation of thrombin was determined as the cleavage of the specific chromogenic substrate. Controls containing active thrombin alone, FII alone, or lacking FII (blank) were used. The values represent the mean ± standard deviation of triplicates and are representative of two independent experiments.

**Figure 5:**
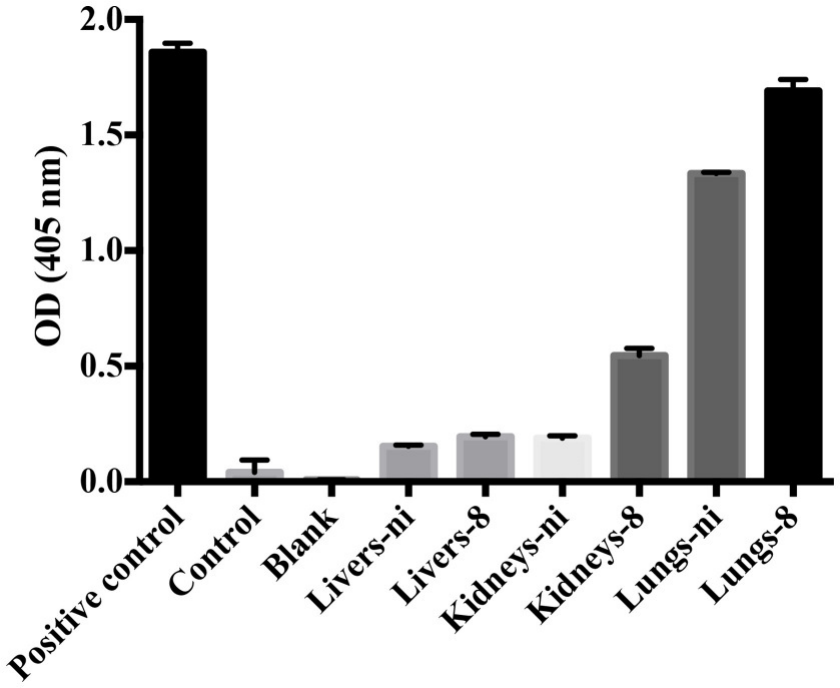
**Ability of leptospirosis organ extracts on the activation of FX and formation of the prothrombinase complex**. Lungs, kidneys, and livers extracts of *Leptospira*-infected hamsters at day 8 or non-infected (ni.) animals were mixed with FVa and PCPS in buffer containing calcium. FXa was omitted from the mixture. A positive control containing FXa was used. In addition, a control lacking FXa as well as a blank reaction lacking FII were also employed. After 30 min incubation at 37°C, FII was added and its conversion to thrombin was measured as cleavage of the chromogenic substrate (*OD* = 405 nm) after 16 h. The values represent the mean ± standard deviation of triplicates and are representative of two independent experiments.

In the next experiments, we assayed the ability of organ extracts from infected animals to activate FII. When lungs from ni. and lungs and kidneys from infected hamsters day 8 were mixed with FII, the formation of thrombin, measured by the substrate hydrolysis, was observed to similar ODs of the ones obtained in the prothrombinase complex assay (shown in Figure 3) only after 16 h incubation (Figure 4). We hence conclude that the organs from *L. interrogans* infected animals contain a factor able to activate FII, which activity is accelerated in the prothrombinase complex. Therefore, we performed the prothrombinase complex assay omitting FXa from the mixture, to evaluate if any FXa-like could be responsible for the FII activation or a thrombin-like responsible for substrate hydrolysis. A minor FXa- or thrombin-like activity was observed. The substrate hydrolysis ODs similar the ones obtained in the full prothrombinase complex assay (shown in Figure 3), were obtained only after 16 h incubation (Figure 5), denoting a slow activation of the prothrombinase complex.

**Figure 6:**
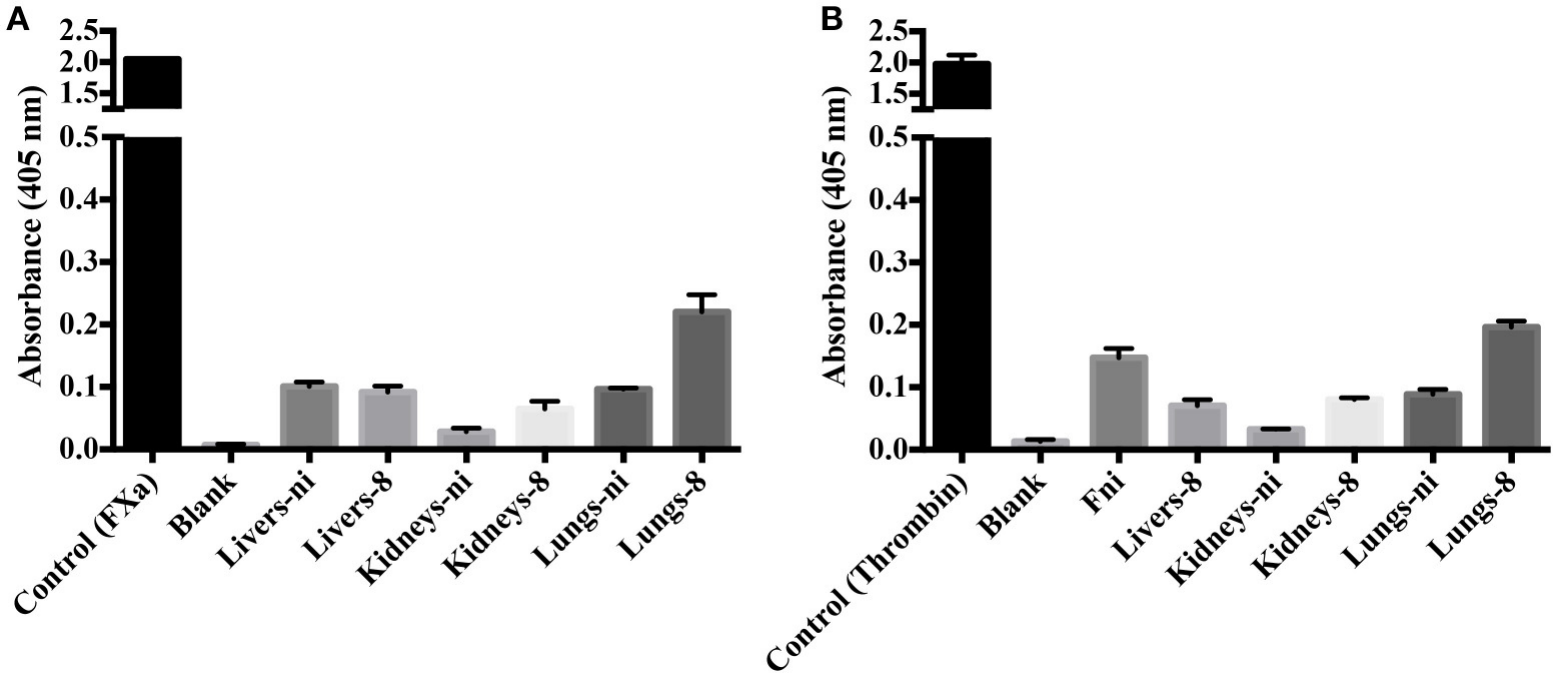
**Activity of leptospirosis organ extracts on the cleavage of FXa and thrombin-specific substrates**. Lungs, kidneys, and livers extracts of *Leptospira*-infected hamsters at day 8 or non-infected (ni.) animals were mixed with the substrates S-2765 **(A)** or S-2238 **(B)** in buffer containing calcium for thrombin and FXa activity, respectively. Controls containing FXa and thrombin were used. After 16 h incubation at 37°C, the cleavage of the chromogenic substrates was measured. The values represent the mean ± standard deviation of triplicates and are representative of two independent experiments.

**Figure 7:**
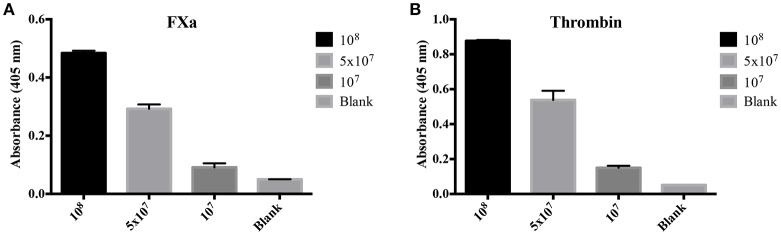
**Activity of *L. interrogans* on the cleavage of FXa and thrombin-specific substrates**. *L. interrogans* extracts (representing final concentration of 10^8^, 5 × 10^7^ or 10^7^ cells/mL) were mixed with the substrates S-2765 **(A)** or S-2238 **(B)** in buffer containing calcium for thrombin and FXa activity, respectively. Controls lacking leptospires were used (blank). After 16 h incubation at 37°C, the cleavage of the chromogenic substrates was measured. The values represent the mean ± standard deviation of triplicates and are representative of two independent experiments.

To evaluate the origin of the FXa- or thrombin-like activities observed (whether from the host or bacteria), we assayed both organ extracts from infected animals and *L. interrogans* extracts for FXa and thrombin activities by specific chromogenic substrates. The organs from infected hamsters (livers, kidneys, and lungs) resulted in low FXa (Figure 6A) and thrombin activities (Figure 6B) even after 16 h incubation, while the bacterial extracts occasioned dose-dependent and significant FXa (Figure 7A) and thrombin activities (Figure 7B). We conclude by these experiments that leptospires were responsible for FXa and thrombin-like coagulation factors activities that were detected in the previous assay (see Figure 5).

### The assembly of the prothrombinase complex may occur at the leptospiral surface

**Figure 8:**
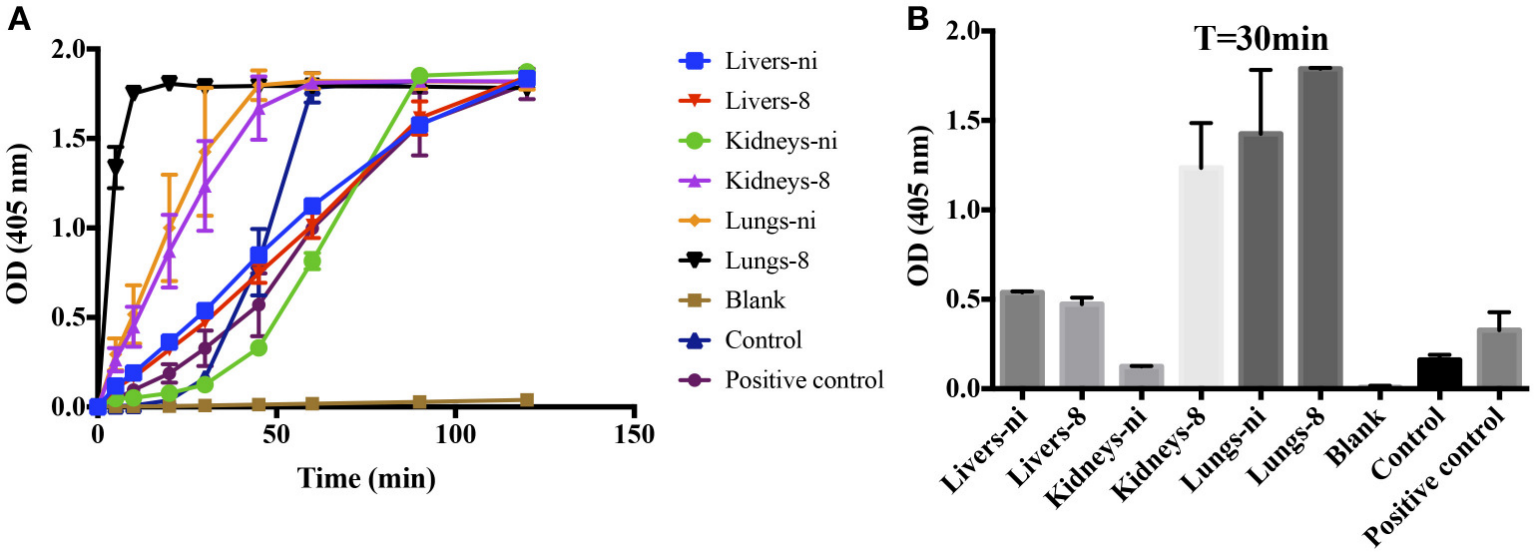
***L. interrogans*** **cells role as phospholipidic surface for the assembly of the prothrombinase complex**. Lung, kidney, and liver extracts of *Leptospira*-infected hamsters at day 8 or non-infected (ni.) animals were mixed with FXa, FVa, and leptospires as source of phospholipidic surface in buffer containing calcium. A positive control lacking leptospires and containing PSPC was used. After 30 min incubation at 37°C, FII was added and its conversion to thrombin was measured as cleavage of the chromogenic substrate (*OD* = 405 nm) at different time points **(A)**. In **(B)**, the bars represent the absorbance obtained 30 min after FII addition. The values represent the mean ± standard deviation of triplicates and are representative of two independent experiments.

We have previously shown that leptospires can function as a surface for the contact system activation, binding to coagulation factors (Vieira et al., 2016). When we performed the prothrombinase complex assay by substituting the PSPC by live leptospires, a strong activation of the complex was observed (Figures 8A,B—control), showing that the leptospires membrane could play a similar role and act as a phospholipidic surface for the prothrombinase complex assembly. For comparison purposes, we performed the prothrombinase complex in the presence of PSPC (positive control). Additionally, when the organ extracts were added to the prothrombinase complex mixture using leptospires as a surface for assembly, we observed the acceleration of the reaction (Figure 8A), similar as observed when PSPC was used (see Figure 3).

### Hypothesis of leptospiral interference with the host hemostasis

**Figure 9:**
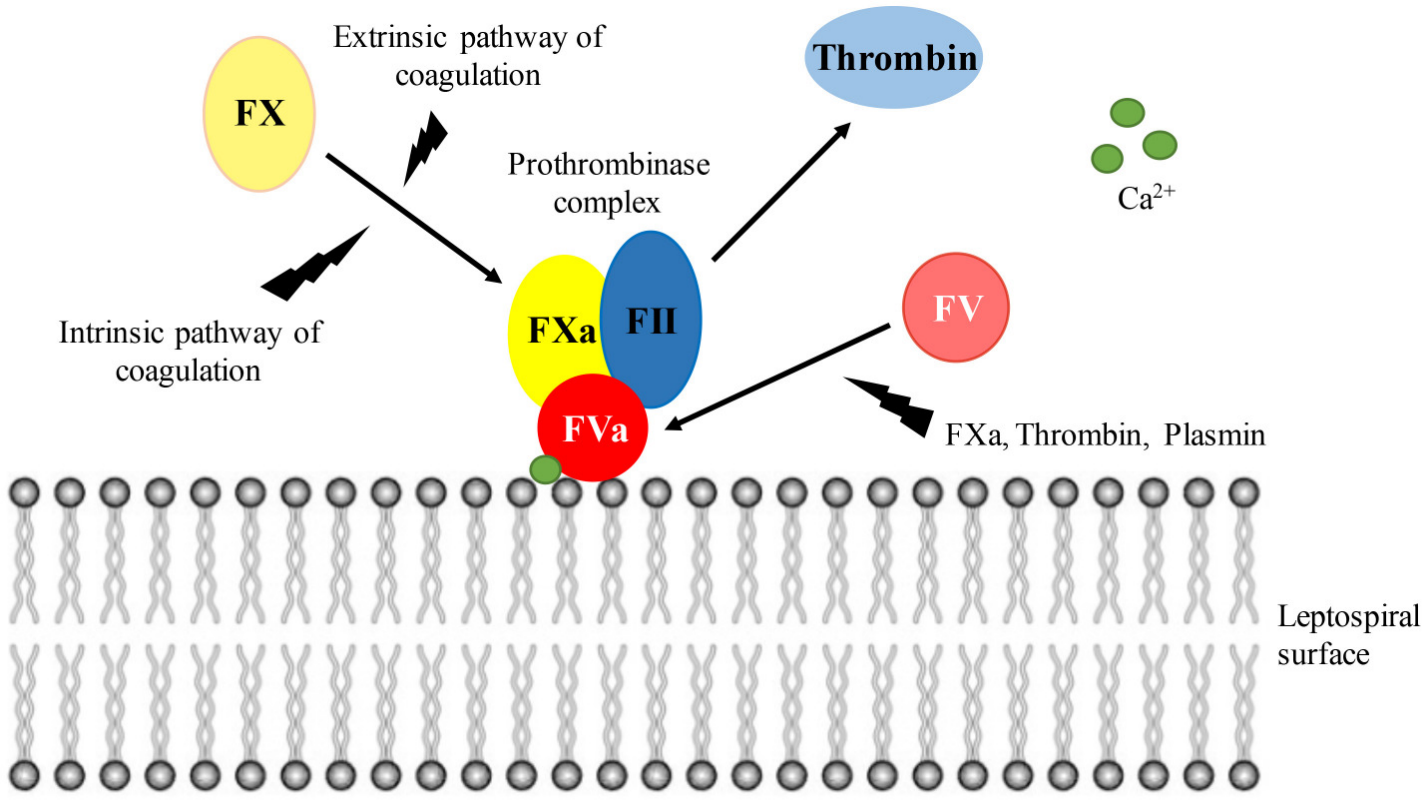
**Prothrombinase complex assembly at the leptospiral surface**. In the presence of calcium ions, FVa binds to the leptospiral surface, acting as a receptor for FII and FXa. Within the prothrombinase complex, thrombin formation is augmented. Both the intrinsic and extrinsic pathways of coagulation lead to FX activation. The cofactor FV can be activated by FXa, thrombin, and plasmin.

Figure 9 depicts the binding and activation of the prothrombinase complex at the leptospiral surface. In the presence of calcium ions, the cofactor FVa binds to the leptospiral outer membrane surface, recruiting the coagulation factors FII (prothrombin), and FXa. Although thrombin can be generated in solution via activation by FXa in the presence of calcium ions, the thrombin formation is accelerated more than 300,000 times within the prothrombinase complex assembled at a phospholipidic surface (Rosing et al., 1980; Betz et al., 1997). FX can be activated by either the intrinsic (contact system) and extrinsic (tissue factor) pathways of coagulation. FXa, thrombin, and plasmin may act as a source for FV activation.

## Discussion

Bleeding is a common symptom of severe leptospirosis. Pathological findings reveal widespread hemorrhages at mucous membranes, peritoneum, muscles, and organs such as kidneys and lungs (Arean, 1962; De Brito et al., 2013). The intense intra-alveolar hemorrhage with interstitial inflammatory infiltrates and vascular extravasation seems to be unique for leptospirosis (Pereira et al., 2005; De Brito et al., 2013). However, the cause and mechanisms of bleeding are still not clearly elucidated. Accumulating data indicate that compromised hemostasis might be involved in the manifestation of leptospirosis hemorrhagic syndromes. Evidence point toward activation of coagulation, impaired anticoagulation, and activation of fibrinolysis (Sitprija et al., 1980; Edwards et al., 1986; De Francesco Daher et al., 2002; Chierakul et al., 2008; Wagenaar et al., 2010; Vieira et al., 2013).

We have previously shown that both human intrinsic and extrinsic pathways of coagulation are modulated in response to *Leptospira* or leptospiral secreted proteins, promoting host pro-coagulant, and pro-inflammatory state, especially in the early phase of the disease (Vieira et al., 2016). We revealed the important role of tissue factor and tissue factor-bearing microvesicles in the induction of the systemic pro-coagulant state during leptospirosis. We have also described the interaction of *Leptospira* with the fibrinolytic system by capturing human plasminogen on its surface and stimulating an imbalance of the normal fibrinolysis by enhancing the availability of plasminogen activators and plasmin generation (Vieira et al., 2009, 2011, 2013; Vieira and Nascimento, 2016).

In the present investigation, we analyzed the coagulation status of experimentally infected hamsters' organ extracts. We aimed to detect coagulation interferences and the possible origin of leptospirosis hemorrhagic symptomatology. We produced a lethal infection that mimicked the clinical presentation of severe leptospirosis in patients and animals (Hartman et al., 1986; Faine, 1999; Yang et al., 2006; Abgueguen et al., 2008; Daher et al., 2010). The focus of our study was on the liver, kidneys, and lungs of experimentally infected hamsters, as they have the highest bacterial burden in leptospirosis, and frequently present hemorrhagic foci and organ failure in severe infection (Faine, 1999).

In the infected hamsters, there was a rapid migration of *Leptospira* to the liver, and to a lesser extent to the kidneys and lungs, in which the bacterial load progressively increased. After that, we noted the gradual clearance of the leptospires from the lungs and livers, while bacteria apparently persisted on the kidneys, as previously reported (Ko et al., 2009; Coutinho et al., 2014). The progression of the disease was associated with tissue pathologies and marked alterations of liver, kidney, and lung structures. All the organs exhibited hemorrhage foci from day 6 after infection.

The presence of the organ extracts prolonged the plasma global coagulation tests (PT, aPTT, and TCT). These results can be originated by inhibition of the intrinsic, extrinsic, and/or common coagulation pathways, a consumption coagulopathy or hyperfibrinolysis. The higher contents of prothrombin F1+2 fragments in the infected animals' organ samples further suggest coagulation activation and ongoing coagulation factors consumption. Prolonged clotting times were shown in human leptospirosis patients, and PT prolongation seems to be associated with both clinical bleeding and poor outcome (Chierakul et al., 2008; Wagenaar et al., 2010).

At the final steps of coagulation, FII can generate thrombin by two pathways: (i) in the presence of FXa and calcium ions or (ii) in the presence of FXa, FVa, and calcium ions in a phospholipidic surface—the prothrombinase complex (Colman et al., 1994; Betz and Krishnaswamy, 1998; Yang et al., 2008; Qureshi et al., 2009). Formation of the full prothrombinase complex is supported by both intrinsic and extrinsic pathways of coagulation, and may enhance the conversion of FII to thrombin by 300,000-fold (Rosing et al., 1980; Betz et al., 1997). We observed a noticeable acceleration of the prothrombinase complex formation when lungs and kidneys extracts of the infected hamsters were added. The organ extracts were only slightly effective in the activation of FII. This was also observable by the prothrombinase complex activity when FXa was omitted, resulting in slow acceleration of the complex. Altogether, our results suggest the presence of a FXa-like factor capable of activating FII or a thrombin-like factor competent in hydrolyzing the substrate in the leptospirosis organ extracts. Additionally, the activity of those factors is accelerated in the prothrombinase complex assembled on a phospholipidic surface.

We reveal that the FXa- and thrombin-like activities originated from the bacteria rather than from the organs. This could be either by *Leptospira* endogenous molecules or leptospiral capture of hosts factors. Indeed, we have previously shown that leptospires bind a number of host coagulation factors from the intrinsic and common pathways of coagulation (FXII, FXa, thrombin; Fernandes et al., 2015; Vieira et al., 2016). The assembling and activation of the contact system at the bacterial surface leads to an activation of the up-stream coagulation factors. Moreover, leptospires induce prolonged aPTT and PT in human plasma *in vitro* by depletion of coagulation factors bound to their surface (Vieira et al., 2016). Therefore, the observed activities are probably due to *Leptospira* interaction with host molecules.

The cofactor FV can be activated by FXa, thrombin, and plasmin. Within the prothrombinase complex, FVa binds to the negatively charged phospholipidic surfaces acting as a receptor for FII and FXa. Here, we show that leptospires can act as a surface for the prothrombinase complex assembly. To our knowledge, this is the first time such bacterial ability is described. Our previous results showed that binding of plasmin (Vieira et al., 2009) and FXa (Vieira et al., 2016) to the leptospiral surface denote various mechanisms by which these bacteria can activate the coagulation cascade. By recruiting host molecules such as plasmin, contact system factors and prothrombinase complex factors to the bacterial surface, leptospires create a very effective environment for the activation of host coagulation and modulation of hemostasis.

Studies demonstrate that the coagulation system is strongly activated in patients with leptospirosis (Wagenaar et al., 2010). The induction of high levels of circulating pro-coagulant microvesicles in the host can induce a systemic pro-coagulant state (Vieira et al., 2016). Additionally, the assembly and activation of the contact system and prothrombinase complex at the leptospiral surface, as well as binding of plasmin, can lead to local and systemic coagulation and fibrinolysis activation (Vieira et al., 2009, 2013, 2016). Based on our results presented here and the data available in the literature, it is suggested that the leptospirosis hemorrhagic syndromes are possibly caused by consumption coagulopathies and hyperfibrinolysis.

Our results add new information regarding *Leptospira* infection. However, we are aware of some limitations. Besides being one of the most studied and used animal model for leptospirosis (Haake, 2006), hamsters are not isogenic, so variable individual responses are expected—and that is why we analyzed pooled samples. Moreover, the leptospires dose we used to inoculate the animals may not reflect the burden acquired during natural infection. However, based on a survival curve and LD50 calculation, we had to stipulate a lethal dose in which all the animals would die within ~10 days post infection. Another drawback is the route of administration. Although the intraperitoneal inoculation is not the natural infection route for leptospires, it is the standard method employed for animal challenge experiments (Haake, 2006; Coutinho et al., 2014).

In any event, we believe that our results further contribute to the understanding of the leptospirosis pathophysiological mechanisms, showing new mechanisms by which the pathogen can interfere with the host hemostasis.

## Author contributions

Conceived and designed the experiments: MV, SD. Performed the experiments: MV, SD, ZM, MD. Analyzed the data: MV, SD, MD. Contributed with reagents/materials/analysis tools: SV, MD, AN. Wrote the paper: MV, AN.

### Conflict of interest statement

The authors declare that the research was conducted in the absence of any commercial or financial relationships that could be construed as a potential conflict of interest. The reviewer MVG and handling Editor declared their shared affiliation and the handling Editor states that the process nevertheless met the standards of a fair and objective review.
